# Ephrin-B2 reverse signaling regulates progression and lymph node metastasis of oral squamous cell carcinoma

**DOI:** 10.1371/journal.pone.0188965

**Published:** 2017-11-30

**Authors:** Eri Sasabe, Ayumi Tomomura, Riki Tomita, Shinya Sento, Naoya Kitamura, Tetsuya Yamamoto

**Affiliations:** Department of Oral and Maxillofacial Surgery, Kochi Medical School, Kochi University, Kohasu, Oko-cho, Nankoku-city, Kochi, Japan; University of North Carolina at Chapel Hill School of Medicine, UNITED STATES

## Abstract

Oral squamous cell carcinoma (OSCC) is a common malignant tumor of the head and neck and frequently metastasizes to cervical lymph nodes. Aggressive local invasion and metastasis of OSCC are significant factors for poor prognosis. In this study, we investigated whether ephrin-B2 expressed in OSCC contributed to tumor progression and lymph node metastasis. Clinical specimens from patients with OSCC had robust ephrin-B2-positive tumor cells and ephrin-B2 protein level was associated with clinical stage, lymph node metastasis, and poor survival outcomes. We also determined that ephrin-B2 protein level was increased in OSCC cell lines compared to normal human oral keratinocytes and that its levels were associated with the migratory and invasive potential of OSCC cell lines. Transfection of an *EFNB2*–specific small interfering RNA (siRNA) into SAS-L1 cells significantly reduced proliferation, attachment, migration, and invasion through phosphorylation of the epidermal growth factor receptor, FAK, ERK1/2, p38, AKT, and JNK1/2 pathways. Furthermore, knockdown of *EFNB2* significantly suppressed adhesion and transmigration of SAS-L1 cells toward human lymphatic endothelial cells. In addition, the growth rate of tumor xenografts and cervical lymph node metastases of OSCC were suppressed by local injection of *EFNB2* siRNA. These results suggest that ephrin-B2 overexpression and activation of the ephrin-B2 reverse signaling pathway in tumor microenvironment in OSCC facilitates progression and lymph node metastasis via enhancement of malignant potential and interaction with surrounding cells.

## Introduction

Oral squamous cell carcinoma (OSCC) has a significant recurrence rate and metastasizes to cervical lymph nodes in approximately 40% of patients with oral cancer [1]. The presence and extent of cervical lymph node metastasis are indicators of disease progression and poor prognosis and must be controlled to improve treatment outcomes [2]. Despite recent developments in prevention and multimodality treatments, OSCC is still characterized by poor prognosis and a low survival rate [3,4]. One of the underlying reasons is the complicated metastasis mechanism, which is a wide-ranging process that includes detachment of cells from tumor tissue, regulation of cell motility, and invasion, proliferation, and evasion through the lymphatic system or blood vessels [5].

Ephrin-B2 is a membrane-anchored ligand for the Eph family of receptor tyrosine kinases (RTKs). An intriguing feature of Eph/ephrin signaling is that both the receptors and the ligands can transduce a signaling cascade following cell-cell interactions, which results in activation of bidirectional signaling pathways. Eph-activated signaling is termed forward signaling, whereas ephrin-activated signaling is termed reverse signaling. Ephrin-B2 also signals in a cell-autonomous fashion and therefore acts independently of Eph receptor interaction [6]. In tumors, ephrin-B2 is widely expressed in blood vessels and involved in tumor angiogenesis as well as neovascularization via promotion of vascular endothelial precursor cell adhesion to the tumor site [7]. Ephrin-B2 is also involved in lymphangiogenesis through induction of vascular endothelial growth factor receptor (VEGFR)-2 and VEGFR-3 uptake by human lymphatic endothelial cells (HLECs), via endocytosis following the activation of VEGFR downstream signaling proteins such as Rac1, Akt, and ERK [8,9]. Importantly, ephrin-B2 was shown to mediate invasion, migration, and angiogenesis in melanoma and glioma cells [10,11]. Several groups showed that the level of ephrin-B2 was significantly increased in head and neck squamous cell carcinoma (HNSCC) and that there was a correlation between elevated ephrin-B2 protein level and poor prognosis [12–15]. However, how ephrin-B2 regulates the behavior of OSCC remains unknown.

In the present study, we investigated the relationship between ephrin-B2 protein level and clinical factors in different cohorts of OSCC patients using immunohistochemistry (IHC). Furthermore, we explored the role of ephrin-B2 in OSCC cells during tumor development and progression using *in vitro* and *in vivo* assays and found that overexpression of ephrin-B2 in OSCC cells and activation of ephrin-B2 reverse signaling pathway in the tumor microenvironment facilitated progression and lymph node metastasis via augmentation of malignant potential and interaction of OSCC cells with surrounding cells.

## Materials and methods

### Cell culture and patient samples

OSCC cell lines established at our laboratory as well as the SAS-L1 OSCC cell line, which is a green fluorescent protein (GFP)-labeled highly lymph node metastatic tongue squamous cell carcinoma (a gift from Dr. Shintani at Showa University), were cultured in Dulbecco's Modified Eagle's medium (DMEM) supplemented with 10% (v/v) fetal bovine serum (FBS) (Invitrogen, Carlsbad, CA, USA) [16,17]. Primary human keratinocytes (PHK) (JCRB Cell Bank, Osaka, Japan) and cells from the immortalized human oral keratinocyte cell line RT-7 (a gift from Dr. Kamata at Hiroshima University) were cultured in Keratinocyte-SFM (Gibco BRL, Gaithersburg, MD, USA) [18]. HLECs (ScienCell Research Laboratories, Carlsbad, CA, USA) were cultured in endothelial cell medium (ScienCell Research Laboratories). Recombinant human ephrin-B2/Fc and Eph-B4/Fc were purchased from R&D Systems (Minneapolis, MN, USA). The Fc fragment of human IgG and the anti-human IgG Fc antibody were purchased from Jackson ImmunoResearch (Baltimore, MD, USA). Before treatment, each Fc was clustered by preincubation with the anti-human IgG Fc antibody at a ratio of 1:2 on ice for 2 h. *EFNB2*–specific small interfering RNA (siRNA) was synthesized by Ambion (Austin, TX, USA). Transfection was performed with Lipofectamine RNAiMAX Transfection Reagent (Invitrogen).

OSCC samples were obtained from surgically excised tissue specimens of 50 patients (Table 1). Written informed consent was obtained from all patients in accordance with the guidelines of the Ethics Committee on Medical Research of Kochi Medical School.

**Table 1:** Associations between ephrin-B2 protein level and clinicopathological factors.

		Ephrin-B2		
	n	Low	High	*p* value
Age (Years)				
<60	16	6	10	1.000
≥60	34	13	21	
Sex				
Male	32	11	21	0.552
Female	18	8	10	
Site				
Tongue	41	19	22	0.029
Cheek	3	0	3	
Mouth floor	6	0	6	
T classification				
T1	10	6	4	0.417
T2	21	8	13	
T3	11	3	8	
T4	8	2	6	
N classification				
N0	25	17	8	<0.001
≥N1	25	2	23	
Stage				
I	10	6	4	0.003
II	11	8	3	
III	11	3	8	
IV	18	2	16	
Clinical type				
Superficial	1	1	0	0.529
Exophytic	23	9	14	
Endophytic	26	9	17	

### Cell proliferation assay

Cell proliferation assays were performed with the Cell Counting Kit-8 (CCK-8; Dojindo Laboratories, Kumamoto, Japan). OSCC cells lines were seeded in 96-well culture plates (5 × 10^3^ cells/well) and cultured for 24 h. SAS-L1 (5 × 10^3^ cells/well) transfected with *EFNB2* or control scrambled siRNA were incubated with 2 μg/mL clustered Fc, ephrin-B2/Fc or Eph-B4/Fc. At indicated time points, absorbance (450 nm) of reduced WST-8 was measured by a microplate reader (Tecan Sunrise).

### Apoptosis assay

SAS-L1 cells were transiently transfected with *EFNB2* siRNA or control scrambled siRNA and cultured for 24 h. Next, the cells were incubated with 2 μg/mL clustered Fc, ephrin-B2/Fc or Eph-B4/Fc. At indicated time points, cells were stained with propidium iodide and FITC-conjugated annexin V (Sigma-Aldrich, St. Louis, MO, USA) and analyzed on a FACScan cytometer using CELLQUEST (Becton Dickinson, San Jose, CA, USA).

### Adhesion assay

A total of 2 μg/mL clustered Fc, ephrin-B2/Fc or Eph-B4/Fc was precoated in 96-well plates at 4°C overnight. Next, nonspecific binding sites were blocked with 3% (w/v) bovine serum albumin (BSA) in phosphate-buffered saline (PBS) at 37°C for 1 h. After three washes with PBS, 1 × 10^5^ control or *EFNB2* siRNA-transfected cells were plated and cultured at 37°C for 30 min. After three washes with PBS, adherent cells were fixed with 4% (w/v) paraformaldehyde and stained with 0.5% (w/v) crystal violet at room temperature for 10 min. Crystal violet in stained cells were extracted by 200 μL dimethyl sulfoxide, and absorbance was measured at a wavelength of 570 nm by a microplate reader (Tecan Sunrise).

### Invasion assay

The invasive potential of cells was evaluated using a BioCoat Matrigel Invasion Chamber kit (Becton Dickinson Biosciences, Bedford, MA, USA). Briefly, OSCC cell lines and SAS-L1 cells transfected with *EFNB2* or control scrambled siRNA (7.5 × 10^4^/500 μL) were added to the transwell insert chamber containing a filter coated with Matrigel. In the lower compartment, 750 μL DMEM containing 10% FBS was used as the chemoattractant. SAS-L1 cells were incubated with 2 μg/ml clustered Fc, ephrin-B2/Fc or Eph-B4/Fc in DMEM. After 22 h, the inserts were removed, and noninvading cancer cells remaining on the upper side of the filter were scraped off. Cells that invaded the lower side of the filter were then stained with the Diff-Quick solution and microscopically observed and counted in five viewfields at ×200 magnification.

### Migration assay

Migratory potential of cells was examined using CytoSelect^™^ 24-well cell migration assay (Cell Biolabs, San Diego, CA, USA). OSCC cells lines and SAS-L1 transfected with *EFNB2* or control scrambled siRNA were seeded in 24-well plates containing proprietary treated plastic inserts at 2.5 × 10^5^ cells/well and cultured for 24 h. The inserts were then removed, and the cells were cultured in the presence of clustered Fc, ephrin-B2/Fc or Eph-B4/Fc for 10 h. After staining, the percentage of closure of the wound field by light microscopy was determined.

### Immunofluorescence and confocal microscopy

LabTek^TM^ chambers were precoated with 2 μg/mL clustered Fc, ephrin-B2/Fc or Eph-B4/Fc at 4°C overnight, followed by incubation with 2 × 10^4^ cells at 37°C for 4 h. After washing with PBS, adherent cells were fixed with 4% (w/v) paraformaldehyde for 20 min and permeabilized with 0.1% (v/v) Triton X-100 for 10 min. Actin filaments were stained with rhodamine-phalloidin (1:1000, Invitrogen) for 45 min. After washing with PBS, the chambers were mounted using an antifade mounting fluid containing DAPI (4',6-diamidino-2-phenylindole), and images were captured using a Fluoview FV-1000D confocal laser scanning microscope (Olympus, Tokyo, Japan).

### Western blot analysis

Cells were scraped into RIPA buffer (50mM Tris pH 7.4, 150mM NaCI, 1mM EDTA, 0.25% sodium deoxycholate, 1.0% NP-40, and protease inhibitors), and lysates were separated by sodium dodecyl sulfate-polyacrylamide gel electrophoresis and transferred onto Immobilon-P membranes (Millipore, Bedford, MA, USA). Blocking was performed in Tris-buffered saline containing 5% (w/v) skim milk powder and 0.1% (v/v) Tween-20. Membranes were probed with antibodies to ephrin-B2 (Transduction Laboratories, Lexington, KY, USA) and to β-actin (Santa Cruz Biotechnology, Santa Cruz, CA, USA). Detection was performed using an ECL system (GE Healthcare).

### Quantitative real-time reverse transcription-polymerase chain reaction

SAS-L1 cells were transiently transfected with 75 pmol *EFNB2* siRNA and cultured for 24 h. The cells were then cultured in the presence of 2 μg/mL clustered Fc, ephrin-B2/Fc or Eph-B4/Fc for 24 h. Total cellular RNA was extracted using the RNeasy total RNA isolation system (Qiagen, Valencia, CA). The extracted RNA (1 μg) was added to 20 μL reverse transcription buffer (50 mM Tris-HCl pH 8.3, 50 mM KCl, 10 mM MgCl_2_, 1 mM EDTA, 10 μg/mL bovine serum albumin, and 1 mM dithiothreitol [DTT]) containing 10 mM dNTP, 50 U RNase inhibitor, 1μg oligo dT-primer, and 50 U avian myeloblastosis virus reverse transcriptase (all from Takara Biomedicals, Kyoto, Japan). The reaction mixture was incubated at 42°C for 40 min and heated at 99°C for 5 min. Quantitative real-time reverse transcription-polymerase chain reaction was performed using a master mix provided in the TaqMan universal PCR kit and the ABI PRISM 7000 sequence detection system (Applied Biosystems, Foster City, CA, USA). Primers for *EFNB2* mRNA and the TaqMan probes were obtained from Applied Biosystems. Expression level of the endogenous reference gene human β-actin was determined using the commercially available TaqMan predeveloped assay reagent (Applied Biosystems).

### Proteome profiler array

To identify ephrin-B2–regulated signal transduction proteins, we used the Proteome Profiler™ Human Phospho-Kinase Array kit (R&D Systems). Densitometric analysis of the arrays was performed using the TotalLab TL100 image analysis software (Nonlinear Dynamics, Newcastle upon Tyne, UK).

### Capacity of SAS-L1 cells to adhere to human lymphatic endothelial cells

HLECs (2 × 10^4^ cells) were seeded in 96-well plates and cultured for 72 h. Once HLECs reached confluency, 1 × 10^5^ SAS-L1 cells transfected with *EFNB2* or control scrambled siRNA were cocultured in the presence of clustered Fc for 1 h. After removal of nonattached cells by washing with PBS, the attached GFP-positive SAS-L1 cells were counted using an All-in-One Biorevo BZ-9000 fluorescence microscope (Keyence Japan, Osaka, Japan).

### Transendothelial migration assay

Transendothelial migration assay was performed using the CytoSelect™ Tumor Transendothelial Migration Assay kit (Cell Biolabs). Briefly, HLECs (1 × 10^5^ cells) were cultured on porous inserts (8 μm) in 24-well plates for 72 h. SAS-L1 cells transfected with *EFNB2* or control scrambled siRNA were labeled with CytoTracker™ and placed over the HLEC monolayer in the presence of the clustered Fc. After 24 h, non-migrated cells in the inner chamber were removed with cotton-tipped swabs. The cells that migrated outside the inner chamber were collected and resolved in lysis buffer. Fluorescence intensity was measured with a Fluoroskan Ascent FL fluorescence plate reader (Thermo Lab systems, Helsinki, Finland) at excitation and emission wavelengths of 485 and 520 nm, respectively.

### Xenograft tumor model

Mice were maintained at the Institute for Laboratory Animal Research, Kochi Medical School. All experimental procedures performed were approved by the Institutional Animal Care and Use Committee of Kochi Medical School (Permit Number: F-00108). The completed ARRIVE guidelines checklist is provided in S1 and S2 Tables. All scientist participating in this work had adequate training in animal care and handling before beginning animal work. Upon arrival, mice were randomized into experimental groups and housed as four animals per cage with free access to food and water under specific pathogen-free conditions. Body weight and overall condition of all animals were monitored daily, and mice were euthanized if they exhibited physiologic signs of stress due to inability to eat or drink, weight loss, or if the tumor volume exceeded 10% of total body weight. None of the mice became severely ill or died throughout the experiment. Only male mice were used to exclude potential hormonal influences such as ovarian cycles in females.

For the xenograft tumor model, SAS-L1 cells were subcutaneously injected into the dorsal flank region of 5-week-old male BALB/c nu/nu mice (Japan Clea, Osaka, Japan). *EFNB2* siRNA and atelocollagen complexes were administered to mice using the AteloGene Local Use *in vivo* siRNA transfection kit (Koken, Tokyo, Japan). *EFNB2* siRNA was injected every four days for a total of ten times into the back of mice under sodium pentobarbital anesthesia, and all efforts were made to minimize suffering. Tumor size was measured with calipers, and tumor volume was calculated every four days as follows: (length × width^2^) × 0.5. All mice were euthanized by carbon dioxide inhalation in a dedicated chamber on day 40.

The incidence of spontaneous tumor metastases was reported to be higher in SCID mice than in age-matched nude mice [19]. Therefore, we transplanted SAS-L1 cells into the tongue of SCID mice to examine OSCC metastasis for evaluation of cervical lymph nodes. SAS-L1 cells were orthotopically injected into the tongue of 5-week-old male SCID mice, and *EFNB2* siRNA was injected into the tongues of mice every three days, for a total of four times, under sodium pentobarbital anesthesia. All mice were euthanized by carbon dioxide inhalation in a dedicated chamber on day 19.

### Immunohistochemistry and scoring system

All samples were fixed with 10% (v/v) formalin, embedded in paraffin, and cut into 4-μm-thick sections. Antigens were retrieved using the HistoVTOne solution (Nacalai Tesque, Kyoto, Japan). The sections were incubated overnight at 4°C with a primary antibody to ephrin-B2 (Atlas Antibodies AB, Stockholm, Sweden), followed by incubation with the EnVision horseradish peroxidase-labeled polymer (Dako, Glostrup, Denmark) for 30 min and development with 3, 3 -diaminobenzidine (DAB) solution for 5 min. The sections were counterstained with Mayer’s hematoxylin. Results were classified according to two parameters based on a previously described modified method [20]: extent of ephrin-B2 staining (score: 0, no staining; 1, <35% positive cells; 2, 35%–75% positive cells; 3, >75% positive cells) and staining intensity (score: 0, no staining; 1, 2, and 3: weak, moderate, and strong staining, respectively). By multiplying the staining extent with the intensity, the following IHC staining grades were obtained: grade 0, no staining; grade 1–2, weak staining (+1); grade 3–4, moderate staining (+2); grade 6–9, strong staining (+3). Finally, for statistical comparisons, specimens with no (0) or weak staining (+1) were classified as the low ephrin-B2 group, whereas those with moderate (+2) or strong (+3) staining were classified as the high ephrin-B2 group.

### Statistical analysis

Results were expressed as means ± standard error of means. Differences were compared using Mann–Whitney's *U* test for continuous variables and Fisher’s exact test for categorical variables. The Kaplan–Meier model was used to evaluate survival rates. All statistical analyses were performed using the EZR software package (Saitama Medical Center, Jichi Medical University, Saitama, Japan), which is a graphical user interface for R (The R Formulation for Statistical Computing Version 3.0.3) [21]. Statistical significance was defined as a p value <0.05.

## Results

### Immunohistological examination of clinical specimens from 50 patients with OSCC

**Fig 1:**
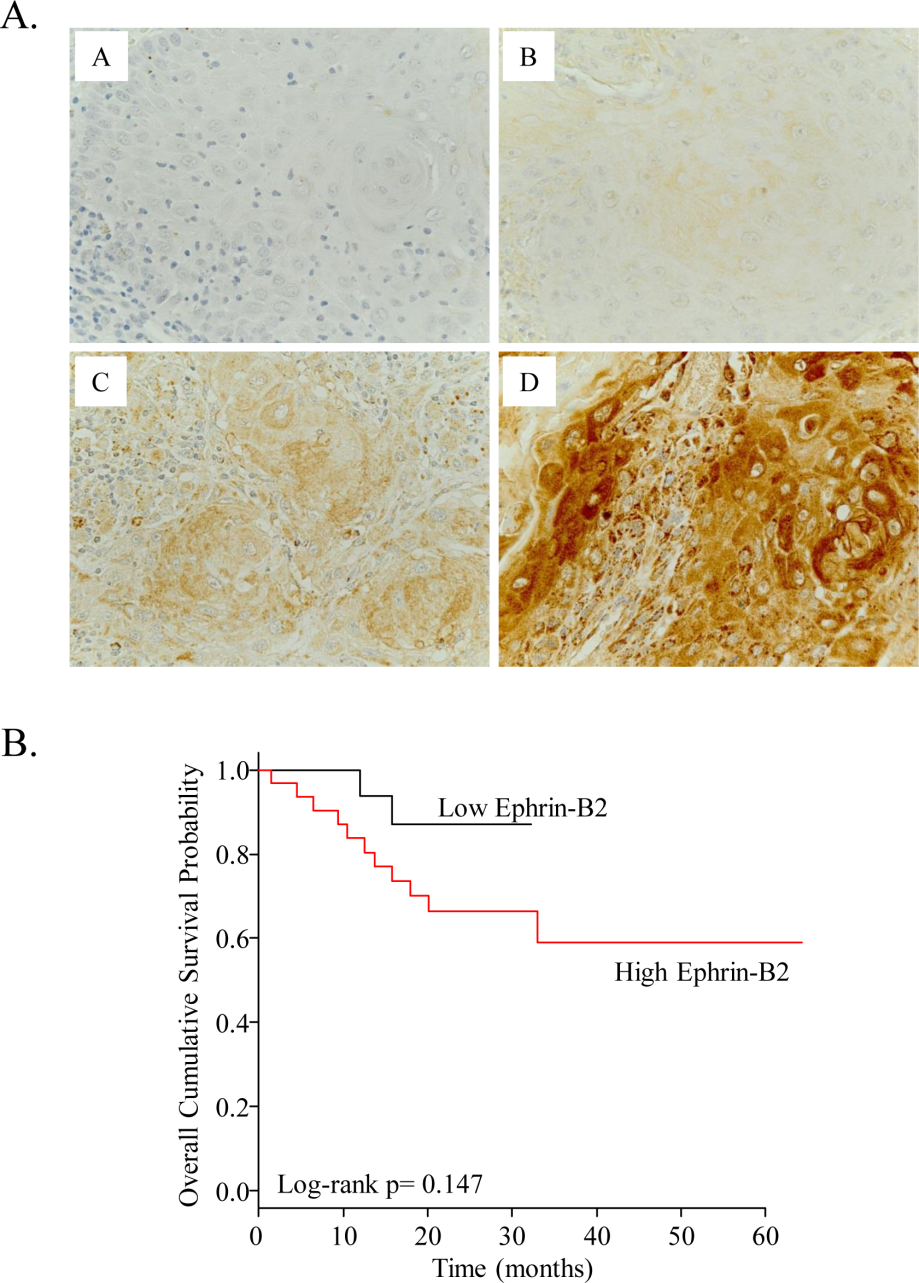
Immunohistological examination of clinical specimens from 50 patients with invasive OSCC. (**A**) Clinical specimens were immunostained for ephrin-B2, and immunoreactivity was scored in a grading scale (panel A: 0, panel B: +1, panel C: +2, panel D: +3). (**B**) Overall survival rates in the low ephrin-B2 (score of 0 or +1) and high ephrin-B2 (score of +2 or +3) groups were analyzed by the Kaplan–Meier estimate.

Clinical specimens from 50 patients with invasive OSCC were immunostained with ephrin-B2 (Table 1). We focused on the tongue and floor of the mouth, both of which are rich in lymphatics; additionally, tumors in these sites have a higher risk of nodal metastases. In this cohort, 50% of the patients were positive for cervical lymph node metastasis. Representative IHC scoring for ephrin-B2 in invasive OSCC is shown in Fig 1A, in which immunoreactivity was scored as one of four grades between 0 and +3. We found that there was a correlation between ephrin-B2 immunoreactivity and prognosis in patients with invasive OSCC. The high ephrin-B2 group showed a poorer overall survival rate compared to the low ephrin-B2 group (Fig 1B). Furthermore, we found that ephrin-B2 immunoreactivity was significantly correlated with lymph node metastasis (*p* < 0.001) and clinical stage (*p* = 0.003; Table 1). These findings suggested the presence of a relationship between high ephrin-B2 expression and progression/lymph node metastasis.

### The relationship between ephrin-B2 protein level and malignant potential in OSCC cell lines

**Fig 2:**
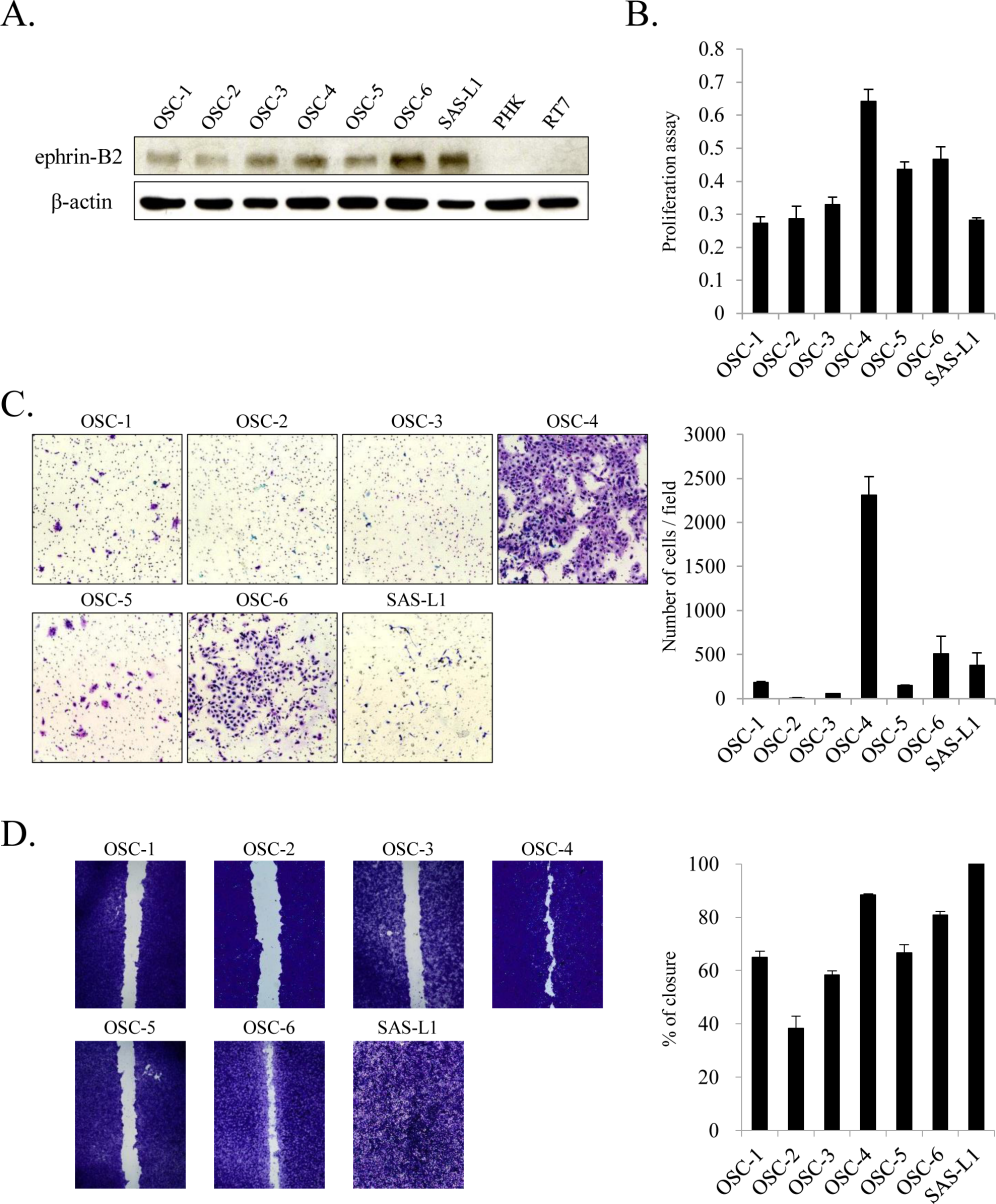
Correlation between ephrin-B2 protein level and malignant potential of OSCC cell lines. (**A**) Total cell lysates were extracted, and ephrin-B2 protein level was determined by western blot analysis. The numbers of (**B**) viable, (**C**) migrating, and (**D**) invading cells were determined using the WST-8, wound healing, and invasion assays, respectively. Values are presented as means ± standard deviation (SD); n = 8 per group.

We next examined whether ephrin-B2 was expressed in OSCC cell lines (OSC-1–OSC-6 and SAS-L1 cells) and PHK and RT-7. We found increased ephrin-B2 protein levels in OSCC cell lines compared to normal human oral keratinocytes. Among OSCC cell lines, protein levels of ephrin-B2 were high in OSC-4, OSC-6, and SAS-L1 cell lines; intermediate in OSC-1, OSC-3, and OSC-5 cell lines; and low in OSC-2 cell lines (Fig 2A). Furthermore, we examined the correlation between malignant potential and ephrin-B2 protein level in each OSCC cell line. We found that OSC-4 was the most proliferative line, followed by OSC-6 and OSC-5, whereas other cell lines exhibited comparable proliferative potential (Fig 2B). In addition, we found that OSC-4 was the most invasive line, followed by OSC-6 and SAS-L1. Although OSC-1, OSC-3, and OSC-5 cell lines exhibited slight invasiveness potential, invasion was limited in OSC-2 cells (Fig 2C). Furthermore, the migratory potential was high in SAS-L1, OSC-4, and OSC-6 cell lines (Fig 2D). OSC-1, OSC-3, and OSC-5 cells showed comparable migratory potential, whereas that of OSC-2 cells was very limited. The OSCC cell lines with higher ephrin-B2 protein levels (OSC-4, OSC-6, and SAS-L1 cells) exhibited more aggressiveness, invasiveness, and migratory potential than the OSCC cell lines with intermediate or low ephrin-B2 protein levels. Overall, these results suggested that ephrin-B2 level was higher in OSCC cells than in human keratinocytes and that ephrin-B2 level was correlated with the malignant potential of tumor cells.

### Effect of *EFNB2* knockdown on proliferation and apoptosis of SAS-L1 cells

**Fig 3:**
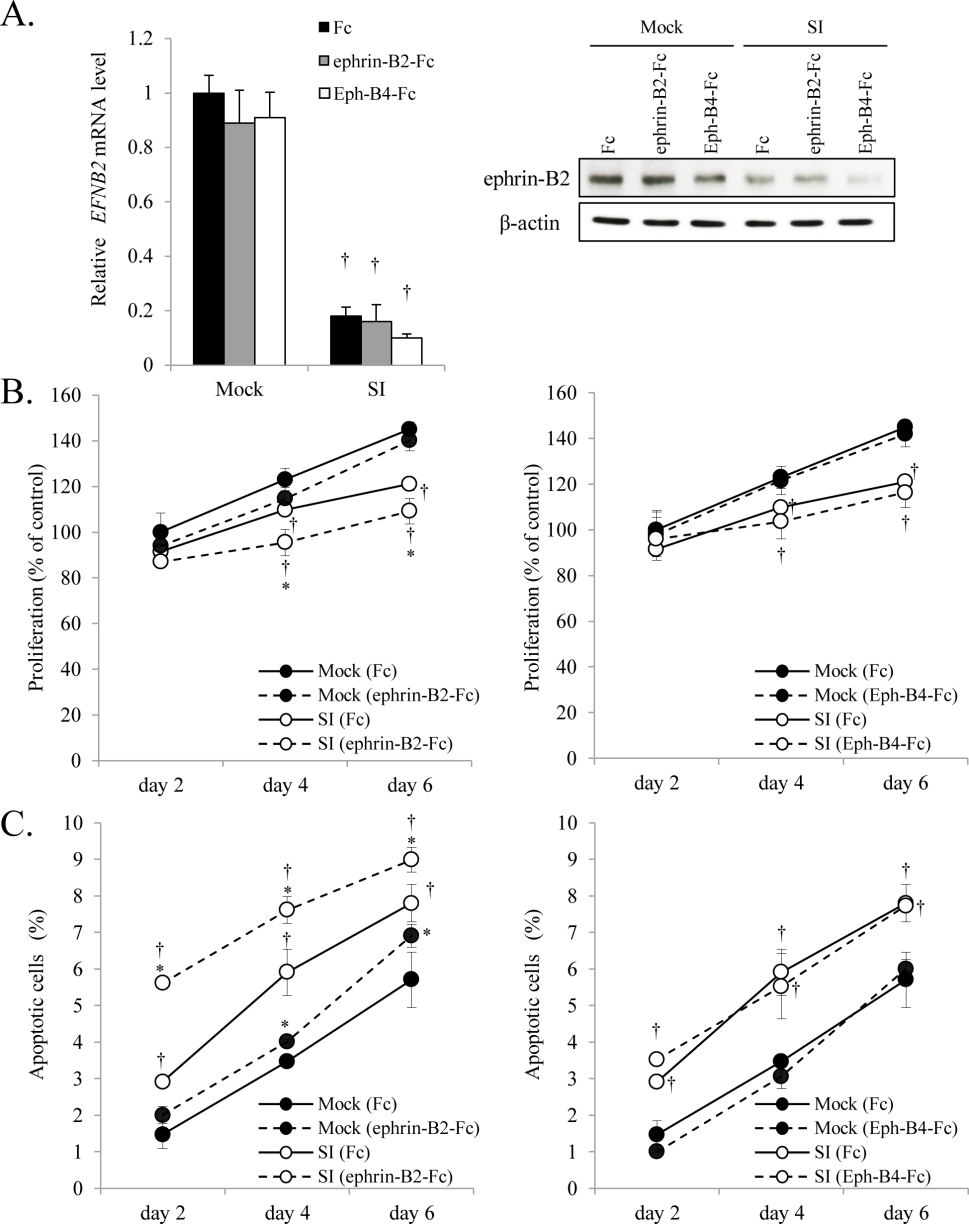
Effect of *EFNB2* knockdown on proliferation and apoptosis of SAS-L1 cells. (**A**) *EFNB2* mRNA expression was assessed by quantitative RT-PCR after 48 h following transient transfected of SAS-L1 cells with 75 pmol *EFNB2* siRNA. Ephrin-B2 protein level was assessed by western blotting. (**B, C**) SAS-L1 cells were transiently transfected with *EFNB2* siRNA and cultured for 24 h. Next, the cells were cultured with 2 μg/mL Fc, ephrin-B2-Fc, or Eph-B4-Fc for 2, 4, or 6 days. After incubation, the numbers of (**B**) viable and (**C**) apoptotic cells were measured by WST-8 assay and flow cytometry, respectively. Values are presented as means ± standard deviation (SD); n = 8 per group. *p < 0.05 compared to Fc-treated cells and ^†^*p* < 0.05 compared to mock-transfected cells.

To examine the effects of endogenous ephrin-B2 on proliferation and apoptosis of OSCC cells, we introduced *EFNB2* siRNA into SAS-L1 cells and confirmed its knockdown at the mRNA and protein levels (Fig 3A). Next, we treated control and *EFNB2* siRNA-transfected SAS-L1 cells with Eph-B4-Fc to verify the effects of ligation of ephrin-B2 with its receptor Eph-B4 and with ephrin-B2-Fc to investigate the effect of exogenous ephrin-B2. We found that cells with *EFNB2*-knockdown showed significantly suppressed proliferation. Exogenous ephrin-B2-Fc also inhibited proliferation in both control and *EFNB2*–silenced cells. In contrast, Eph-B4-Fc chimera did not affect cell proliferation (Fig 3B). Furthermore, we found that apoptosis was augmented in cells with *EFNB2*-knockdown maintained in serum-free medium. Although exogenously added Eph-B4-Fc chimera showed no overt effects, apoptosis of SAS-L1 cells was induced by treatment with ephrin-B2-Fc chimera (Fig 3C). These results suggested that OSCC cells expressing abundant ephrin-B2 were highly proliferative and resistant to serum starvation-induced apoptosis independently of Eph-B4 ligation.

### Effect of *EFNB2* knockdown on attachment, migration, and invasion of SAS-L1 cells

**Fig 4:**
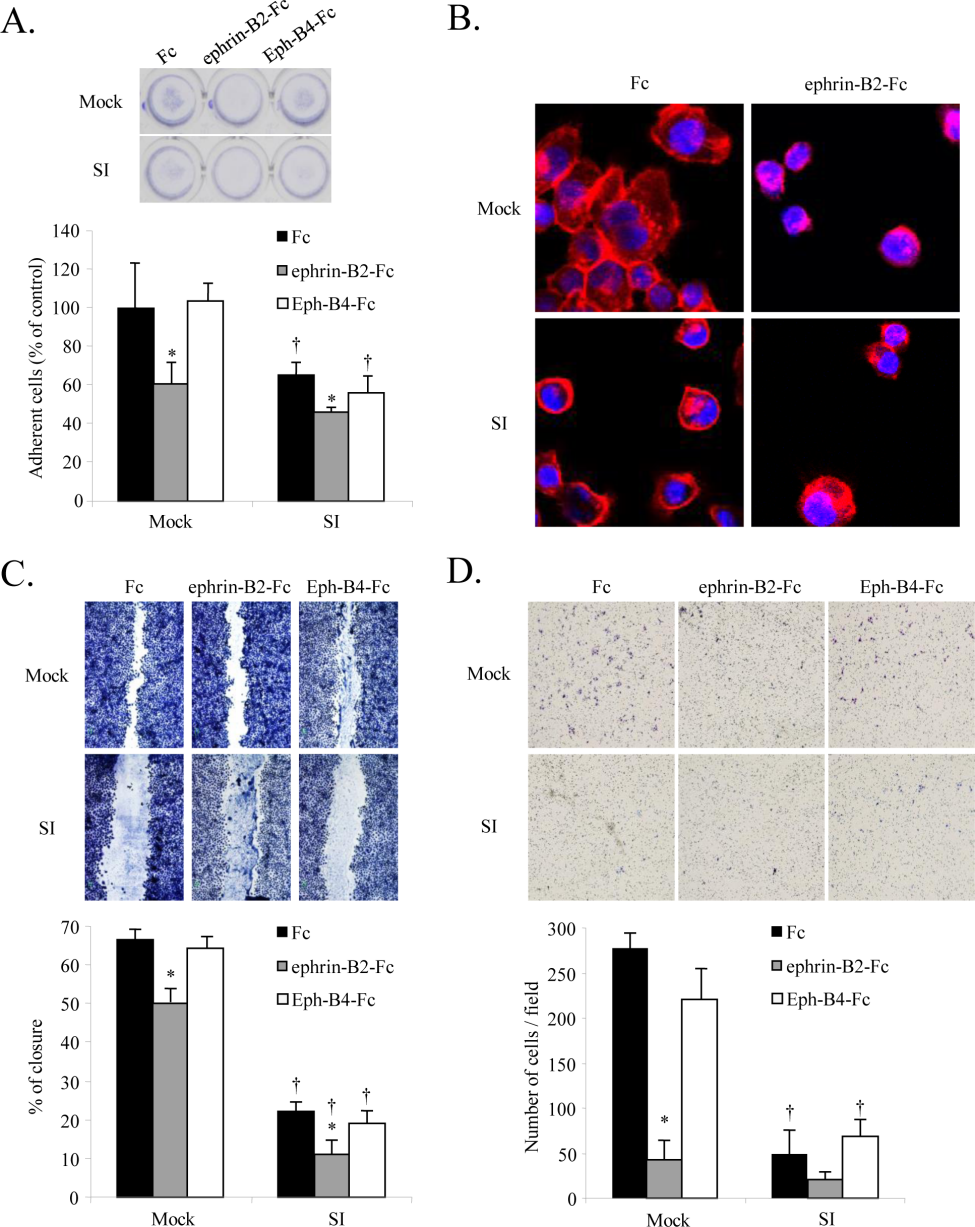
Effect of *EFNB2* knockdown on attachment, migration, and invasion of SAS-L1 cells. (**A**) Control and *EFNB2* siRNA-transfected SAS-L1 cells were seeded in wells precoated with 2 μg/mL Fc, ephrin-B2-Fc chimera, or Eph-B4-Fc chimera for 1 h, and adherent cells were counted. (**B**) Cell morphology was examined by phalloidin-labeled F-actin staining. (**C, D**) The numbers of (**C**) migrating and (**D**) invading cells were determined using wound healing and invasion assays, respectively. Values are presented as means ± standard deviation (SD); n = 8 per group. *p < 0.05 compared to Fc-treated cells and ^†^*p* < 0.05 compared to mock-transfected cells.

Given that the Eph/ephrin system is involved in cell adhesion through bidirectional signal transduction, we examined whether ephrin-B2 was involved in this process in OSCC. We found that *EFNB2* knockdown suppressed cell adhesion in OSCC cell lines compared to control cells. Furthermore, precoating wells with the ephrin-B2-Fc chimera but not the Eph-B4-Fc chimera significantly suppressed adhesion in both control and *EFNB2* knockdown cells (Fig 4A).

Next, we assessed the effect of *EFNB2* knockdown on cell morphology by phalloidin-labeled F-actin staining. We found that control cells were polygonal and flat with stretched actin fibers whereas *EFNB2* knockdown and precoating of the culture dishes with the ephrin-B2-Fc chimera increased the proportion of spherical cells (Fig 4B).

Using a wound healing assay to examine the effect of *EFNB2* knockdown on cell migration, we found that *EFNB2* knockdown and treatment with ephrin-B2-Fc significantly suppressed cell migration. However, precoating of the culture dishes with Eph-B4-Fc exerted no obvious effects (Fig 4C). Similarly, we found that cell invasion was significantly suppressed by either *EFNB2* knockdown or the addition of ephrin-B2-Fc, whereas application of Eph-B4-Fc elicited no effect (Fig 4D). These results suggested that OSCC cells expressing high levels of ephrin-B2 exhibited enhanced adhesion as well as migratory and invasive abilities independent of Eph-B4 ligation. Conversely, exogenous ephrin-B2 might inhibit these capabilities of OSCC cells.

### Effect of *EFNB2* knockdown on phosphorylation of protein kinases in SAS-L1 cells

**Fig 5:**
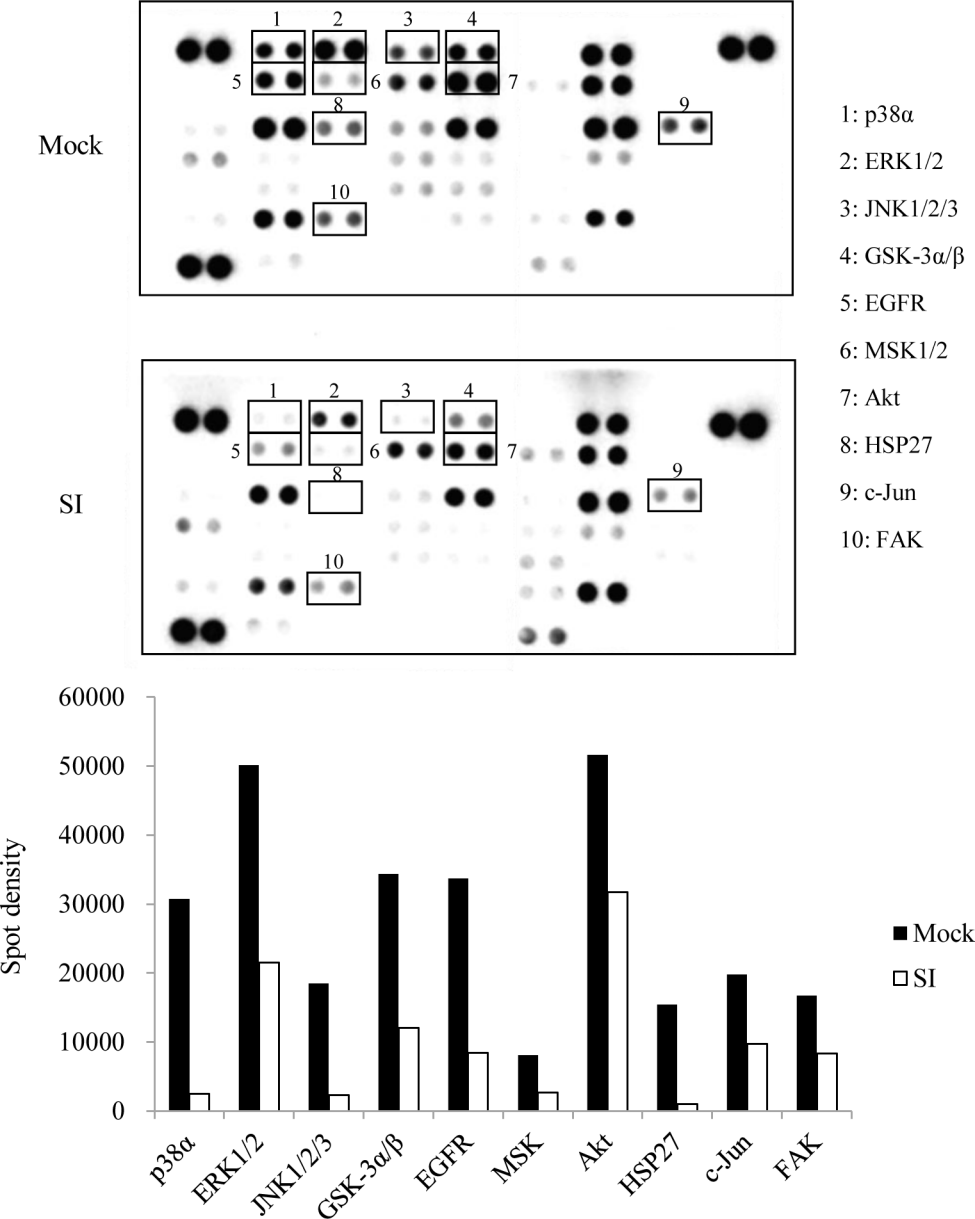
Effect of *EFNB2* knockdown on phosphorylation of protein kinases in SAS-L1 cells. Phosphorylation status of kinases in whole cell lysates from control and *EFNB2* siRNA-transfected SAS-L1 cells was analyzed. Changes in ten representative proteins, which were approximately 40% less hyper-phosphorylated in *EFNB2* knockdown cells compared to control cells, are shown.

To investigate ephrin-B2–activated signal transduction pathways, we compared relative phosphorylation levels of protein kinases in control and *EFNB2*–silenced cells. We found decreased phosphorylation of p38-α (0.08 fold), ERK1/2 (0.43 fold), JNK1/2/3 (0.13 fold), GSK-3α/β (0.35 fold), epidermal growth factor receptor (EGFR) (0.25 fold), MSK1/2 (0.33 fold), Akt (0.60 fold), HSP27 (0.06 fold), c-Jun (0.49 fold), and FAK (0.50 fold) in *EFNB2*–silenced SAS-L1 cells (Fig 5).

### Effect of *EFNB2* knockdown in SAS-L1 cells on tumor growth and lymph node metastasis through interactions with HLECs

**Fig 6:**
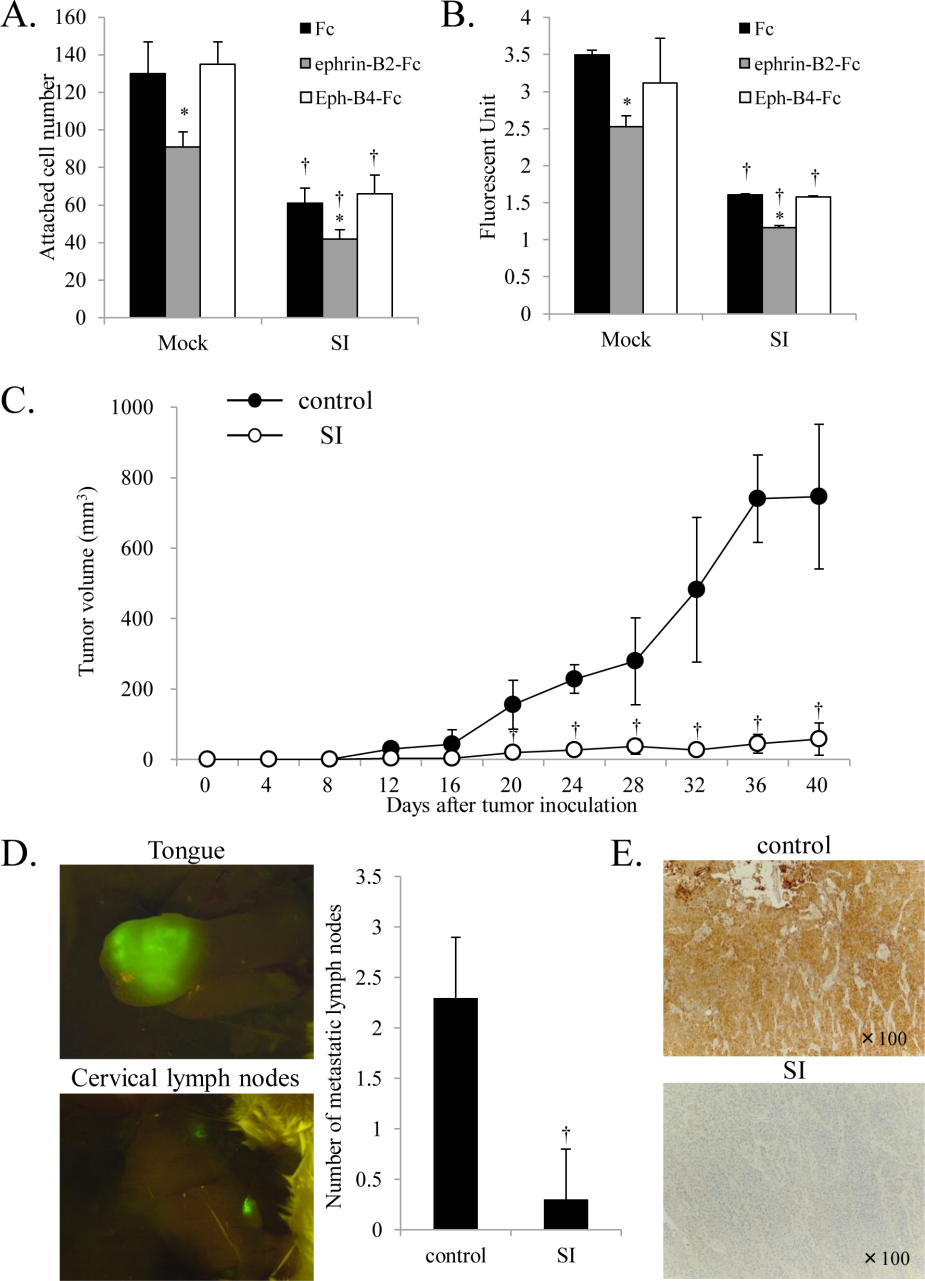
Effect of *EFNB2* knockdown on tumor growth and lymph node metastasis through interactions with HLECs. (**A**) Control and *EFNB2* siRNA-transfected SAS-L1 cells were seeded on HLEC cultures as a monolayer in the presence of 2 μg/mL Fc, ephrin-B2-Fc, or Eph-B4-Fc. GFP-positive cells attached to HLECs were counted. **(B**) Control and *EFNB2* siRNA-transfected SAS-L1 cells were labeled with CytoTracker™ and placed over the HLEC monolayer. After 24 h, SAS-L1 cells that transmigrated toward the HLEC monolayer were lysed, and fluorescence intensity was measured. Values are presented as means ± standard deviation (SD); n = 8 per group. *p < 0.05 compared to Fc-treated cells and ^†^*p* < 0.05 compared to mock-transfected cells. **(C)** BALB/c nu/nu mice were treated as described in the Materials and Methods. Each group contained 8 mice. Volume of xenograft tumors elicited by injection of SAS-L1 cells to the dorsal flank region of mice was assessed for 40 days. Tumor size was determined every 4 days. Values are presented as means ± standard deviation (SD). ^†^*p* < 0.05 compared to control mice. (**D**) SCID mice were treated as described in the Materials and Methods. Each group contained 8 mice. Number of metastatic cervical lymph nodes of control and *EFNB2* siRNA-treated mice was determined. Values are presented as means ± standard deviation (SD). ^†^*p* < 0.05 compared to control mice. (**E**) Ephrin-B2 protein levels in tongues of eight control and *EFNB2* siRNA-treated SCID mice on day 19 was examined by IHC.

To examine the role of OSCC cells expressing ephrin-B2 in lymph node metastasis, the involvement of ephrin-B2 in adhesion and transmigration of OSCC cells toward HLECs was investigated. SAS-L1 cells were seeded on a cultured HLEC monolayer, and GFP-positive cells representing adherent SAS-L1 cells to the HLEC layer one hour later were counted. We found that *EFNB2* knockdown significantly reduced the adhesion ability of SAS-L1. Although treatment with ephrin-B2-Fc suppressed adhesion of SAS-L1 cells to HLECs, treatment with Eph-B4-Fc elicited no appreciable effect (Fig 6A). In addition, transfection of *EFNB2* siRNA into SAS-L1 cells and the addition of ephrin-B2-Fc chimera significantly reduced the number of cells that transmigrated through the HLEC layer (Fig 6B). These results suggested that endogenous ephrin-B2 in OSCC cells promoted adhesion and transmigration toward HLECs independent of Eph-B4 ligation. Conversely, exogenous ephrin-B2 might inhibit these effects.

To further assess the role of ephrin-B2–expressing OSCC cells in tumor growth *in vivo*, we established a tumor mouse model using 5-week-old BALB/c nu/nu mice by subcutaneous injection of SAS-L1 cells into the back of the animals. We found that injection of *EFNB2* siRNA suppressed tumor growth significantly (Fig 6C), which suggested that ephrin-B2 in OSCC cells promoted tumor growth *in vivo*. SCID mice with implanted SAS-L1 cells in the tongue exhibited tumor cell engraftment to the tongue and metastasis to cervical lymph nodes (Fig 6D), whereas injection of *EFNB2* siRNA into the tongue significantly reduced the number of metastatic cervical lymph nodes (Fig 6D). IHC for ephrin-B2 protein levels in the tongues of these mice revealed that the ephrin-B2 protein levels were reduced in mice injected with *EFNB2* siRNA (Fig 6E).

## Discussion

Previous studies showed that ephrin-B2 expression was significantly increased in HNSCC tissue compared to normal tissue [12–14]. Consistent with these findings, we herein showed that ephrin-B2 expression was increased in OSCC cells compared to human keratinocytes. Several ephrins are induced by hypoxia and hypoxia-inducible factor-1α (HIF-1α) [22]. Solid tumors, including OSCC, possess hypoxic areas in their central mass because of a decreased vascular supply and an initially increased energy demand by cancer cells. We previously showed that HIF-1α was overexpressed in OSCC cells and that its expression was induced under hypoxic conditions [23]. Therefore, HIF-1α signaling may act as a trigger for ephrin induction in OSCC. Furthermore, we found that ephrin-B2 expression was correlated with the malignant potential of OSCC cells, suggesting that ephrin-B2 might regulate tumor progression in OSCC.

Additionally, we demonstrated that endogenous *EFNB2* knockdown inhibited proliferation, adhesion, migration, and invasion of OSCC cells. Furthermore, treatment of OSCC cells with an ephrin-B2 ligand, the Eph-B4-Fc chimera, did not influence these processes, suggesting that the cancer-promoting effect of endogenous ephrin-B2 was independent of its binding with eph-B4 expressed in neighboring cells. Conversely, treatment with ephrin-B2-Fc chimera suppressed the malignant potential of OSCC cells, indicating that the knockdown effects of endogenous ephrin-B2 and addition of exogenous ephrin-B2-Fc chimera led to distinct outcomes. We predicted that exogenous ephrin-B2-Fc chimera might act as a negative feedback regulator to downregulate the effects of endogenous ephrin-B2. OSCC is composed of heterogeneous cell populations and OSCC cells overexpressing ephrin-B2 might become dominant through suppression of proliferation, attachment, migration, and invasion of surrounding cells. Previous studies reported that EphB4 overexpressed in HNSCC cells promoted tumor development by stimulating angiogenesis, increasing cancer cell survival, and facilitating invasion and migration [12,24–28]. Rutkowski *et al*. reported that EphB4 induced aggressive phenotypes were restrained in the presence of ephrin-B2, in part by reducing EphB4 protein levels [29]. Overall, these results suggest that the tumor suppressive effects of exogenous ephrin-B2-Fc might be due to Eph-B4 inhibition.

Signaling of clustered ephrins is initiated by recruitment of Src family kinases and phosphorylation of cytoplasmic ephrin-B tyrosines and substrates implicated in downstream signaling [30,31]. In addition to Src kinases, ephrin-B trans-phosphorylation via activation of VEGFR, platelet-derived growth factor receptor, fibroblast growth factor receptor, EGFR, and TIE-2 demonstrates the presence of an important crosstalk between these signaling pathways [32]. Our results showed that phosphorylation of p38-α, ERK1/2, JNK1/2/3, GSK-3α/β, EGFR, MSK1/2, Akt, HSP27, c-Jun, and FAK was absent in *EFNB2* knockdown cells. These signaling cascades are activated by a wide variety of receptors involved in tumor growth, including RTKs, integrins, and ion channels. EGFR, one of RTKs, regulates downstream signaling by different kinases including Rac1, Akt, ERK, mitogen-activated protein kinase, and c-Jun N-terminal kinase (JNK) [8,9]. Therefore, ephrin-B2 might enhance the malignant potential of OSCC through crosstalk with cell surface receptors such as RTKs, thereby regulating downstream signaling. In addition, tight junction proteins such as claudin, metalloproteases (MMPs) including MMP-8, and a disintegrin and metalloprotease (ADAM) proteins were reported to interact with ephrin-B [33–35]. We previously reported that claudin-1 functioned as an intercellular adhesive molecule involved in the migration and invasion of OSCC cells [36]. Phosphorylation of ephrin-B1 induced by interaction with claudin-1 regulates cell-cell contact formation [33]. Thus, it is conceivable that interaction of ephrin-B2 with other factors in the transmembrane of surrounding cells might contribute to OSCC progression.

We also demonstrated that most actin fibers disappeared and migration/invasiveness was suppressed in *EFNB2* knockdown cells. *EFNB2* knockdown also decreased phosphorylation of FAK. FAK is a cytoplasmic non-receptor tyrosine kinase and functionally involves in regulation of adhesion, migration, invasion, proliferation, and survival. FAK associates with integrins or growth factor receptors and, when activated, localizes to cell-matrix contact sites, focal adhesions [37–39]. OSCC cells were shown to express FAK abundantly and that FAK regulated invasive properties of OSCC [40]. Ephrin-B reverse signaling alters the subcellular localization and/or tyrosine-phosphorylation levels of other proteins, including FAK and paxillin, and is involved in cytoskeletal dynamics [41]. Therefore, ephrin-B2 might regulate the malignant potential of OSCC cells through interaction with FAK.

Metastasis to lymph nodes and distant organs is the most significant factor in determining patient prognosis in cancer. Specifically, while metastasis of OSCC to cervical lymph nodes occurs frequently, the most common treatment is lymph node or neck dissection, is highly invasive procedure [1–4]. Therefore, inhibition of lymph node metastasis in OSCC represents a critically important challenge in patient treatment and care. In this study, we found a correlation between ephrin-B2 expression level and lymph node metastasis in biopsy specimens of primary lesions. Furthermore, we found that metastasis to cervical lymph nodes from tongue squamous cell carcinoma was significantly suppressed by local injection of *EFNB2* siRNA *in vivo*. We also observed that silencing *EFNB2* in OSCC cells reduced their adhesion ability to HLECs and attenuated the number of cells that transmigrated through the HLEC layer. Recently, *EFNB2* expression was reported to significantly correlate with overall survival and disease-free survival in HNSCC, as reported by a study which analyzed The Cancer Genome Atlas (TCGA) [14]. These findings suggest that ephrin-B2 expression might be associated with poor survival in OSCC by promotion of lymph node metastasis through facilitating interaction of cancer cells with HLECs.

To date, approaches targeting ephrin-B2 showed potential for cancer treatment. However, many reports suggested that the delay in tumor growth observed by ephrin-B2 targeted therapies might be dependent on angiogenesis inhibition in the tumor. The findings from the current study indicate that ephrin-B2 targeted therapy acts not only on vascular and lymphatic endothelial cells but also directly on cancer cells, thereby additively suppressing tumor progression. Thus, ephrin-B2-targeted therapy might be useful as a prophylactic treatment for lymph node metastasis of OSCC, and if proven effective, in combination with primary treatment.

To conclude, our findings demonstrated that in OSCC cells overexpressing ephrin-B2, activation of the ephrin-B2 reverse signaling pathway in the tumor microenvironment facilitated progression and lymph node metastasis via enhancement of the malignant potential and interactions with surrounding tumor cells. Further investigation is needed to determine whether ephrin-B2 expression in OSCC can predict prognosis in patients and whether antagonism of ephrin-B2 signaling might be an effective therapeutic strategy.

## Supporting information

S1 Table ARRIVE guidelines checklist. (JPG)

S2 Table ARRIVE guidelines checklist. (JPG)
