# Supplementary figures and images for: Ephrin-B2 reverse signaling regulates progression and lymph node metastasis of oral squamous cell carcinoma
Source: PLoS One. 2017 Nov 30;12(11):e0188965. doi: 10.1371/journal.pone.0188965 (PMC5708812; doi:10.1371/journal.pone.0188965)

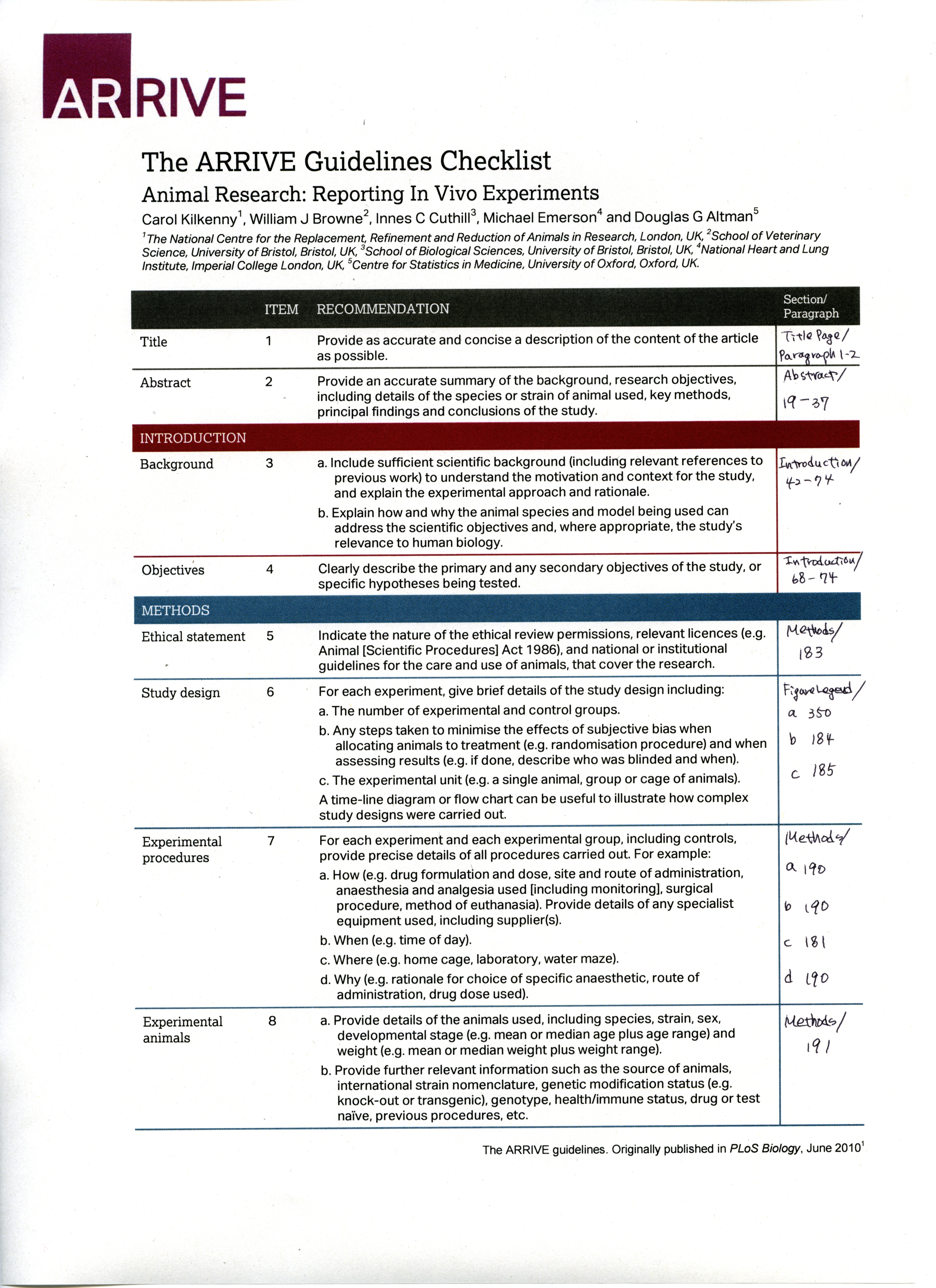

Supplement: S1 Table — (JPG) [file pone.0188965.s001.jpg]

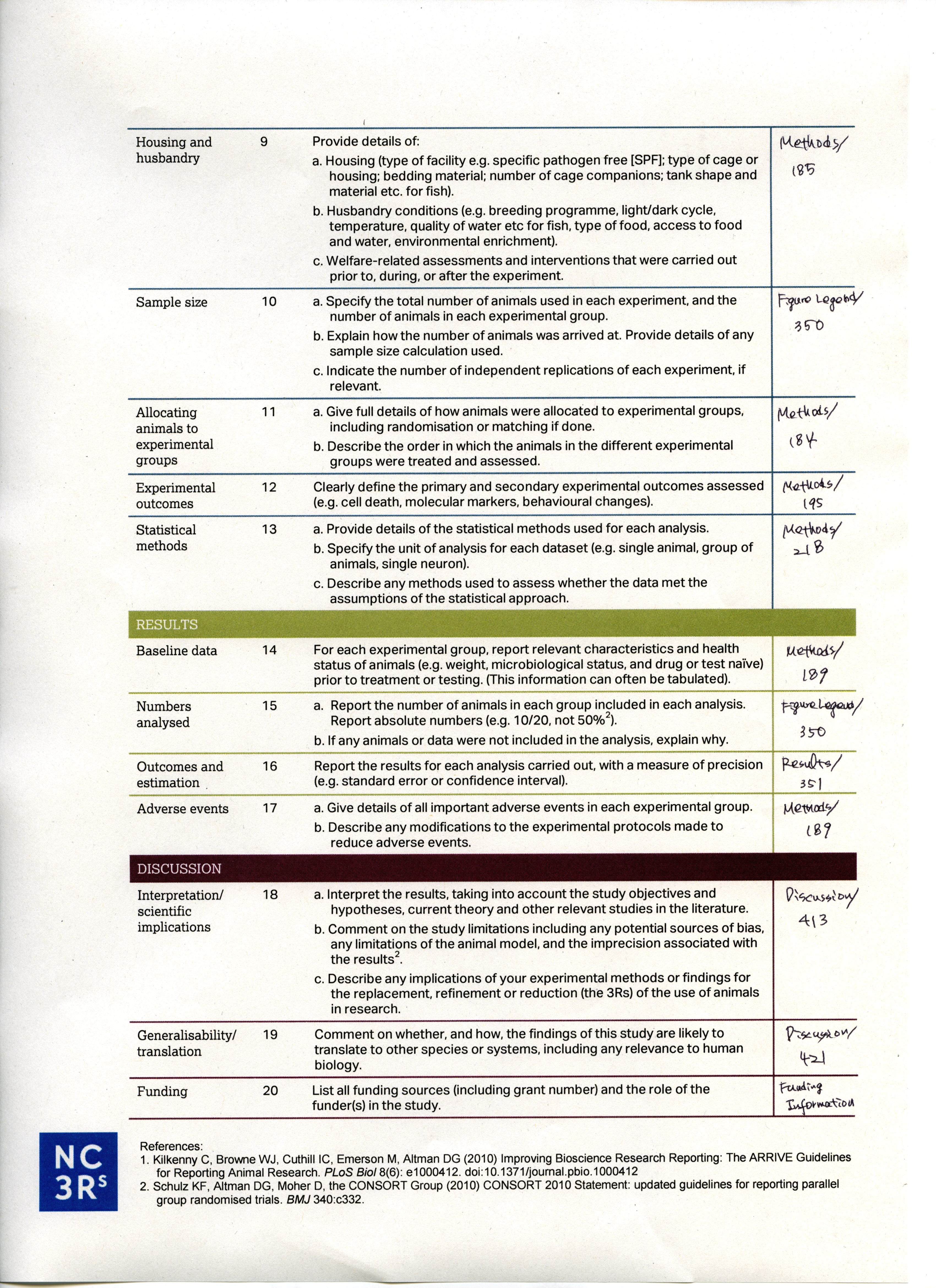

Supplement: S2 Table — (JPG) [file pone.0188965.s002.jpg]
